# Structural connectivity-based predictors of cognitive impairment in stroke patients attributable to aging

**DOI:** 10.1371/journal.pone.0280892

**Published:** 2023-04-14

**Authors:** Barbora Rehák Bučková, David Kala, Jakub Kořenek, Veronika Matušková, Vojtěch Kumpošt, Lenka Svobodová, Jakub Otáhal, Antonín Škoch, Vlastimil Šulc, Anna Olšerová, Martin Vyhnálek, Petr Janský, Aleš Tomek, Petr Marusič, Přemysl Jiruška, Jaroslav Hlinka

**Affiliations:** 1 Department of Complex Systems, Institute of Computer Science of the Czech Academy of Sciences, Prague, Czech Republic; 2 Department of Cybernetics, Faculty of Electrical Engineering, Czech Technical University in Prague, Prague, Czech Republic; 3 National Institute of Mental Health, Klecany, Czech Republic; 4 Department of Circuit Theory, Faculty of Electrical Engineering, Czech Technical University in Prague, Prague, Czech Republic; 5 Department of Developmental Epileptology, Institute of Physiology, Academy of Sciences of Czech Republic, Prague, Czech Republic; 6 Department of Mathematics, Faculty of Nuclear Sciences and Physical Engineering, Czech Technical University in Prague, Prague, Czech Republic; 7 Department of Physiology, Second Faculty of Medicine, Charles University, Prague, Czech Republic; 8 Department of Neurology, Second Faculty of Medicine, Charles University, Motol University Hospital, Prague, Czech Republic; 9 MR Unit, Department of Diagnostic and Interventional Radiology, Institute for Clinical and Experimental Medicine, Prague, Czech Republic; University of Minnesota, UNITED STATES

## Abstract

Despite the rising global burden of stroke and its socio-economic implications, the neuroimaging predictors of subsequent cognitive impairment are still poorly understood. We address this issue by studying the relationship of white matter integrity assessed within ten days after stroke and patients’ cognitive status one year after the attack. Using diffusion-weighted imaging, we apply the Tract-Based Spatial Statistics analysis and construct individual structural connectivity matrices by employing deterministic tractography. We further quantify the graph-theoretical properties of individual networks. The Tract-Based Spatial Statistic did identify lower fractional anisotropy as a predictor of cognitive status, although this effect was mostly attributable to the age-related white matter integrity decline. We further observed the effect of age propagating into other levels of analysis. Specifically, in the structural connectivity approach we identified pairs of regions significantly correlated with clinical scales, namely memory, attention, and visuospatial functions. However, none of them persisted after the age correction. Finally, the graph-theoretical measures appeared to be more robust towards the effect of age, but still were not sensitive enough to capture a relationship with clinical scales. In conclusion, the effect of age is a dominant confounder especially in older cohorts, and unless appropriately addressed, may falsely drive the results of the predictive modelling.

## Introduction

The global burden of stroke is increasing while the disease maintains the second position as the leading cause of deaths and disability, rising to 104.2 million prevalent cases worldwide in 2017 [1]. Despite the drop in incidence in developed countries, the number of cases is growing in low and middle-income states, reinforcing the need to understand the disease and the recovery process better [2].

Stroke is triggered by insufficient blood perfusion of the brain which significantly affects patients and usually leads to considerable sensory-motor and cognitive disabilities [3]. The damage induced by stroke may be direct or indirect—through secondary degeneration.

Sensory-motor impairments following stroke are widely described and include hemibody weakness, skin breaks, urinary tract or chest infections [4–6]. The treatment of muscle-restricted mobility usually consists of various forms of rehabilitation [7, 8]. The field has progressed so far as to construct predictive models to anticipate individual patient motor recovery potential [9, 10].

Contrarily, cognitive comorbidities of acute stroke, which include aphasia, loss of memory, orientation, and attention, although widely prevalent, are not as well understood and treated [11–13]. The current cognitive rehabilitation methods may be thus not optimally targeted [14–17]. As the treatment of post-stroke comorbidities presents a considerable social and economic burden [18], it is necessary to deepen our insight into the structural damage within the affected tissue, primarily the white matter [19]. The loss of white matter integrity is among the most direct consequences of stroke. Research concerning its impact on cognition has so far brought inconclusive results.

One of the well-established methods for studying white matter abnormalities is TBSS applied to Fractional Anisotropy (FA) maps or other white matter integrity metrics, linking localized decrease in FA to the decline in various cognitive scales [20–24].

Another approach to investigate the white matter integrity is to use a structural connectome (SC). Connectome describes brain as a topologically complex interconnected network which balances regional and functional specialisation and integration [25–27]. This results in the coordination of processes across brain regions at low connection cost. However, it also implies that any dysfunction will spread through the network easily, possibly initiating pathological processes [28–30]. SC is determined by the model of white matter fibre pathways that physically connect predefined brain regions and is derived from the diffusion weighted imaging data, using methods of fibre tracking. Quantification of the relationships between the respective units of brain usually leads to the construction of a connectivity matrix which describes the existence and potentially magnitude of interconnection among all parts of the system and may be analysed.

Considering the uncertain effect of white matter integrity on cognition, the reports of the effect of structural disconnection are even more inconclusive. The ambivalence primarily originates in the differences among the study designs as well as in a wide variety of cognitive scales used [31, 32]. Moreover, the added potential of structural connectivity information provided on top of the usual lesion size for outcome prediction was investigated, so far, with contradictory results [33–35].

The analysis of connectivity matrices often employs so-called graph theoretical analysis. In this framework, the connectivity matrix is understood as an adjacency matrix of the graph [36]. The vertices of the graph represent anatomically defined parts of the brain, and the edges are given by the weights of the matrix between individual regions. Graph-theoretical properties quantifying the topological features of the network are then determined. Usually, they include: clustering coefficient, characteristic path length, small-world coefficient, centrality, efficiency, transitivity, assortativity, or rich club coefficient [37–39].

Using these measures individual connectivity profiles may be derived to investigate healthy subjects and provide insights into networks damaged by either functional or structural disconnection. As stroke presents a violent disruption of the healthy network, clinical as well as empirical evidence suggests that investigation of the connectome or its parts could provide new insights into stroke-related comorbidities [40–42].

In summary, the relationship between white matter integrity and possible cognitive impairment following stroke is complex and has not yet been effectively explained. Numerous studies approached the topic using either white matter integrity measures such as fractional anisotropy or, more recently, analysed connectivity networks using structural neural paths derived from tractography [20, 32, 34]. However, the studies are not directly comparable, as they vary in design, methods for quantification and inference concerning white matter integrity disruption, cognitive scales used, and the interval between MRI and cognitive scales measurements with respect to the stroke date.

The time aspect is also of particular importance, as each study may reflect a specific stage of white matter and cognitive recovery. In this work, unlike some previous studies that dealt with the immediate cognitive consequences of stroke, we focus on investigating the degree to which it is possible to predict *future* cognitive status (1 year after stroke) based on the white matter state measured within two weeks after the stroke. This task might be potentially more challenging but, on the other hand, more relevant clinically.

We decided to contribute to the integration of the knowledge in this area by using three different methodologies for investigating the integrity of structural brain connectivity, particularly Tract-Based Spatial Statistics (TBSS), the structural connectivity matrix estimated by tractography, and finally, graph-theoretical analysis thereof. Within each approach, we highlight specific methodological aspects and discuss their role in the analysis and interpretation of the results.

## Materials and methods

### Patients

Patients hospitalised with acute ischemic stroke between October 2015 and March 2017 were considered for the study. Within the acute phase of stroke (sudden onset language impairment, unilateral arm, leg, or face weakness), an appropriate treatment was given (intravenous thrombolysis or/and mechanical thrombectomy) based on the decision of an on-call stroke specialist.

Subsequently, the patients were offered to participate in the study, if they fulfilled the following criteria: age above 18 years, positive supratentorial acute ischemic lesion on admission (confirmed via MRI, in the second week after stroke), and signed informed consent. The exclusion criteria included a history of epilepsy or acute symptomatic seizure preceding the current stroke, antiepileptic drug treatment planned for over two weeks after stroke, history of clinical stroke, and contraindication to gadolinium administration. Furthermore, patients with other neurological (e.g. Alzheimer´s disease, Parkinson´s disease) or psychiatric comorbidities (e.g. bipolar disorder, major depression) possibly affecting congition or brain tissue integrity, were not included. Finally, approximately one year after the stroke, patients underwent a set of neuropsychological tests, which were only administered to patients who did not clinically manifest aphasia.

**Fig 1:**
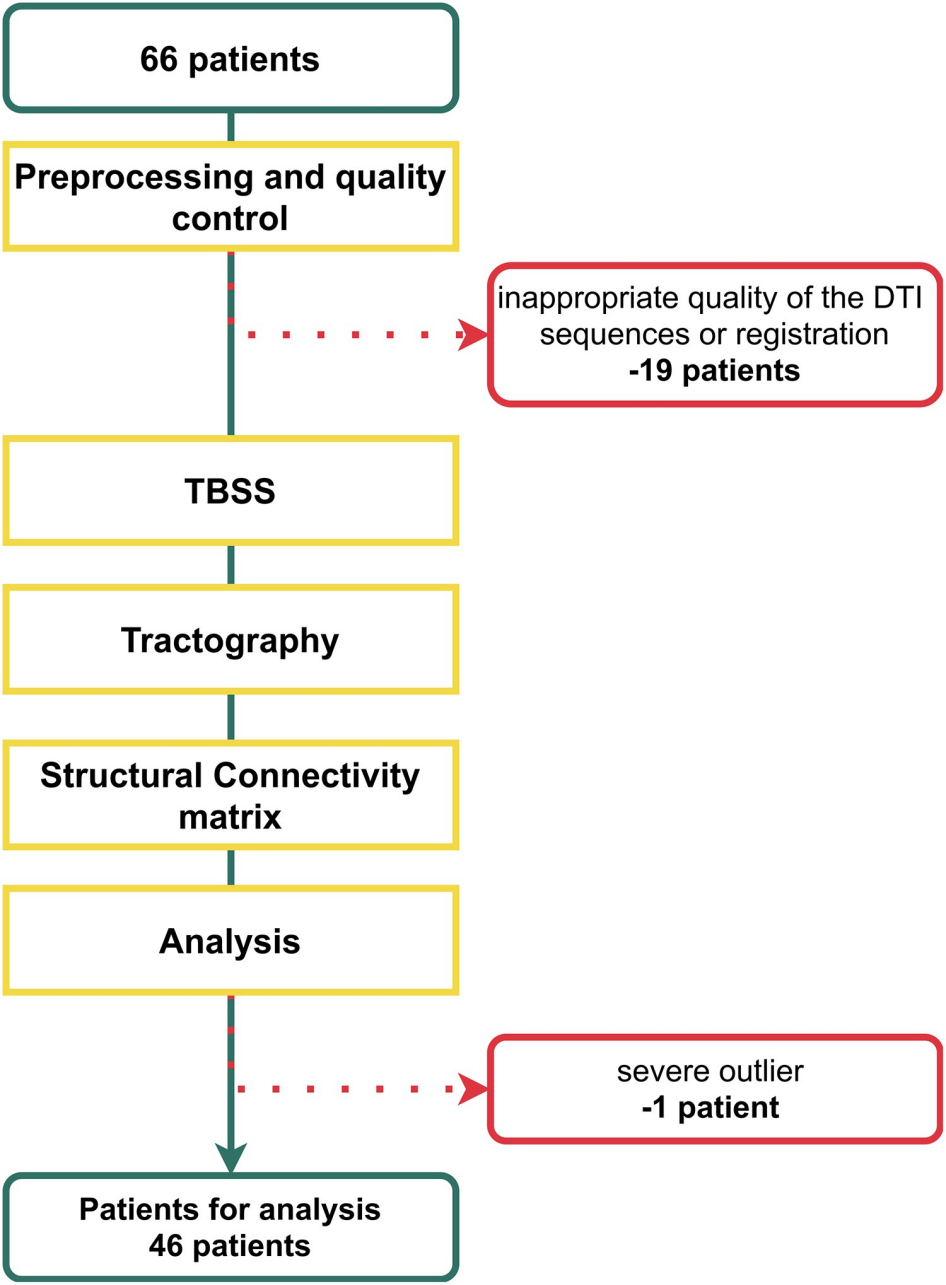
An overview of the analytical process. Initially the data of 66 patients were acquired. The data of 19 patients were discarded because of the unsuitable quality of the diffusion images or unsuccessful image registration. Moreover, we identified one severe outlier in neuropsychological performance who was not included in the analysis. Overall, the data of 46 patients were analysed.

The final dataset included 46 patients fulfilling all criteria (Fig 1, Table 1). All volunteering patients gave written informed consent to participate in the study. Written informed consent was obtained directly from the included patients where possible. The level of information provided to patients was matched to their level of understanding as determined by the investigator. In large hemispheric infarction patients unable to understand or express themselves, the consent was given by a legally authorised representative (e.g., spouse or legal guardian), or physician not participating in the study team, in accordance with regional legal practice and regulations. The study was approved by the Ethics committee of University Hospital Motol (Ref. number: EK-1091/14) and was conducted according to the Declaration of Helsinki ethical principles.

**Table 1:** Description of the sample.

	Men	Women	All
**Number**	24	22	46
**Age (years)**	66 (9; 50; 88)	66 (11; 40; 86)	66 (10; 40; 88)
**Time between measurements (days)**	408 (93; 340; 794)	381 (33; 342; 470)	395 (72; 340; 794)
**Affected hemisphere (R/L/Both)**	10/10/4	12/10/0	22/20/4
**Edinburgh Handedness Inventory**	400 (-150, 400)	400 (-100, 400)	400 (-150, 400)

Age and time between measurements are described as mean (std; min; max). Edinburgh Handedness Inventory is described as median (min; max). Time between measurements stands for the number of days between stroke and cognitive scales measurement.

### Cognitive function assessment

Patients’ handedness was assessed during the acute phase using Edinburgh Handedness Inventory. The rest of the neuropsychological scales were assessed on average 395 days after neuroimaging. The examination lasted approximately 45–60 minutes. Global cognitive performance was assessed by Mini-Mental State Examination (MMSE). The results of the neuropsychological assessment were converted to z-scores and combined into five cognitive domains by averaging the corresponding z-scores. Z-scores of the tests, in which a higher score indicated lower performance (TMT, Prague Stroop Test, BNT) were inverted. The five cognitive domains were as follows: (1) Memory: Free and Cued Selective Reminding Test (Free recall, Total Recall, Delayed free recall, Delayed total recall) [43]; (2) Executive functions: Trail making test part B, Phonemic verbal fluency (letters K,P,S), Similarities from the Wechsler Adult Intelligence Scale-Third Edition, Prague Stroop Test [44, 45]; (3) Attention and working memory: Trail making test Part A, Digit span forward and backward from the Wechsler Adult Intelligence Scale-Third Edition [45]; (4) Language: Boston naming test (15-item version), Semantic verbal fluency (animals) [45]; (5) Visuospatial functions: Visual object and space perception battery (Number location), and Rey-Osterrieth Complex Figure Test [46, 47]. Detailed description of the scores is shown in S1 Table.

### MRI acquisition

MRI imaging was performed seven to twelve days after the onset of the symptoms using 1.5T magnetic resonance scanner (Philips Medical Systems). The acquisition protocol consisted of T1 and T2-weighted anatomical scans, FLAIR contrast, and DWI, with the following parameters: **3D T2 weighted:** TR 3200 ms, TE 263 ms, FA 90°, acquisition matrix 228x227, voxel size in mm 1.1x1.1x1.1; **3D T1 weighted:** TR 25 ms, TE 4.6 ms, FA 30°, acquisition matrix 220x198, voxel size in mm 1.1x1.1x1.1; **DWI:** TR 3157 ms, TE 94 ms, flip angle 90°, acquisition matrix 92x90, acquisition voxel size in mm 2.43x2.49x2.5, the reconstructed matrix dimension: 128x128 resulting in a reconstructed pixel of 1.75x1.75, no gaps, bipolar gradient sampling scheme, b = 0 and 800 s/mm^2^ (one b0 direction and 32 b800 directions).

### Data processing

The diffusion-weighted imaging data were preprocessed using a combination of FSL software [48] and MRtrix3 [49]. First, all images were denoised and Gibbs ringing artefacts were removed using the dwidenoise and mrdegibbs functions, respectively [50–52]. Due to the lack of multiple b0 values we employed the Synb0-DisCo algorithm [53], to synthesize an undistorted non-diffusion weighted image used as an anatomical target for distortion correction. Subsequently, the eddy current correction (eddy) was performed [54] to address geometrical distortions introduced by diffusion acquisition. For the initial analysis, the fractional anisotropy, mean diffusivity (MD), axial diffusivity (AD), and radial diffusivity (RD) maps were generated using the combination of dwi2tensor and tensor2metric commands, and the Tract-Based Spatial Statistics (TBBS) was employed to identify the regions of white matter related to the individual cognition scores [55]. The pipeline non-linearly aligns the fractional anisotropy maps onto a predefined template, and subsequently affine aligns them to a standard MNI space where the image skeleton is created. We applied a threshold of 0.3 onto the mean skeleton, restricting the subsequent analysis only to the most dominant and well-aligned white matter tracts.

The construction of structural connectivity matrices based on deterministic tractography followed. We chose the Tax recursive calibration algorithm to determine the response function (dwi2response) and dwi2fod csd algorithm to estimate the fibre orientation distributions for spherical deconvolution [56, 57]. Deterministic tractography was performed using the tckgen SD STREAM algorithm with the following parameters: number of streamlines: 10 million; step size: 1; the maximum angle in degrees: 60; minimum length (in mm): 10; maximum length (in mm): 300; cutoff for terminating streamline: FA<0.1. [58, 59]. Seeding was performed homogeneously over white matter voxels. The tractograms were inspected, and a disproportional amount of 300 mm long tracts was discovered (as it was the maximum allowed length of the algorithm). To avoid a possible bias caused by these tracts, we decided to discard them. Finally, we registered the Automatic Anatomical Labeling (AAL) atlas [60] in two-step procedure: first, the standard MNI brain was registered to the T1 anatomical image using affine transformation with 12 degrees of freedom (flirt) available in the FSL library. Subsequently, the transformation that registered T1 to the b0 image of diffusion data was performed in the same manner. The individual structural connectivity matrices, contained the absolute count of streamlines between each pair of regions.

### Analysis

In the TBSS part of the analysis, we constructed a general linear model to identify regions of white matter related to each of the cognitive scales. The statistics were corrected using Threshold-free cluster enhancement (TFCE) [61], and 5% level of significance was considered. We also considered variables suspected as potential confounds: age and hemisphere affected by stroke, which we included into the models as covariates.

**Fig 2:**
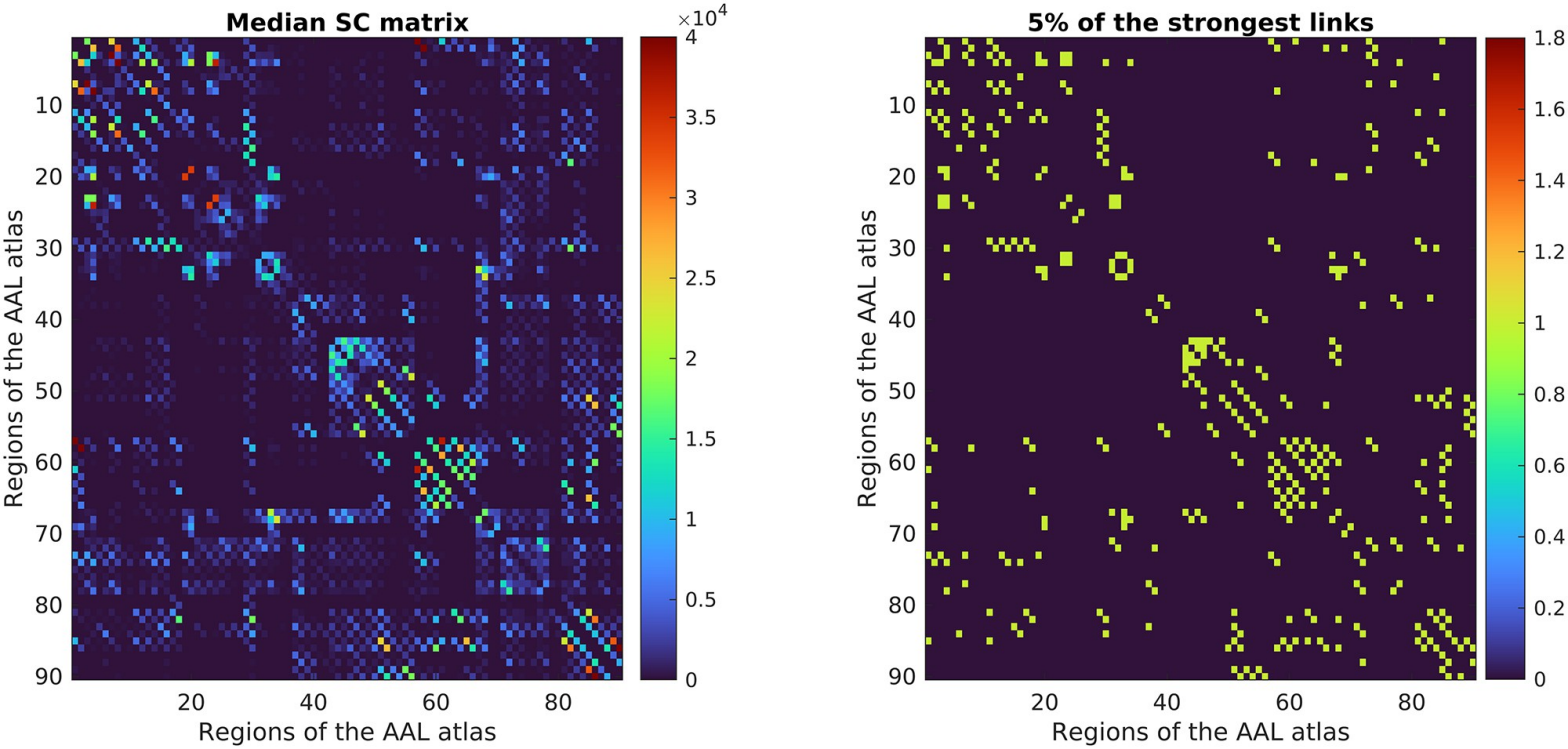
Median Structural Connectivity (SC) matrix. SC containing the median number of streamlines of 46 patients included in the analysis (left) and binary matrix with highlighted 5% of the strongest links (right). Each row/column represents an area of the AAL atlas (in the original order).

In an exploratory analysis of the SC matrices, we initially constructed a median SC matrix, which served as a template defining typical brain structural connectivity. The median was chosen to avoid any bias towards the affected pairs of regions. Based on the template, we selected 5% of the most extensively connected pairs of regions and included them in further analyses (Fig 2). Thus, these strongest links define a kind of a backbone of the most substantial structural connections, including the most relevant 198 out of 3960 possible pairs of regions. Apart from significantly decreasing the amount of connections for which we are going to test, this approach also avoids superfluous analysis of potential false positive edges that might have arisen during tractography. We computed the Spearman correlation (*R*) of selected pairs of regions with age, and each of the six cognitive scales controlled for age. The p-values (*p*) obtained were corrected using False Discovery Rate (FDR) on the level of individual scales [62]. For clarity, we report the raw p-values of this analysis throughout, but only for those pairs of regions, which survived the FDR correction.

Finally, we proceeded with the computation of graph-theoretical measures, which we correlated (using Spearman correlation) with clinical scores. We considered the following measures: assortativity, average strength, clustering coefficient, efficiency, graph energy, characteristic path length, rich-club coefficient, and transitivity. For comprehensiveness, we describe these measures in their binary form. Note that in this work, they have been appropriately adjusted and used in their weighted alternative [63].

*Average strength* is the average of all edge weights in the graph. *Graph energy* is the sum of the absolute values of the eigenvalues of its adjacency matrix. *Assortativity* describes a preference for graph nodes to attach to other similar nodes. Computationally, it is defined as the Pearson correlation coefficient of the node degree between pairs of linked nodes [38], i.e. assessing the connections to nodes similar in terms of connectivity degree or strength.

*Clustering coefficient* of vertex *v* is defined as ratio of all triangles (cycles of length three) around vertex *v* to all possible triangles around *v*. The clustering coefficient of the entire graph is the average over all vertices. It quantifies the tendency of the graph to form clusters and is closely related to *transitivity*–the ratio of 3×number of all triangles in the graph to all possible triangles in the graph.

The *characteristic path length* of vertex *v*, is the average of all the shortest path lengths between the vertex *v* and the remaining vertices of the graph. Subsequently, the characteristic path length of the graph is the average of all characteristic path lengths of the vertices of the graph. Conversely, *efficiency* is an average of the inverse values of the shortest path lengths between all vertices in the graph. It measures the efficiency of information exchange between the vertices.

*ϕ*(*k*) is defined as the ratio of the present number of links to the maximum possible number of links between elements with node-degree at least *k* (in this study, we considered *k* = 70). In other words, *ϕ*(*k*) is the density of the subgraph induced by vertices of degree greater than *k*. Generalisation for weighted graphs is described in detail in [64].

## Results

**Fig 3:**
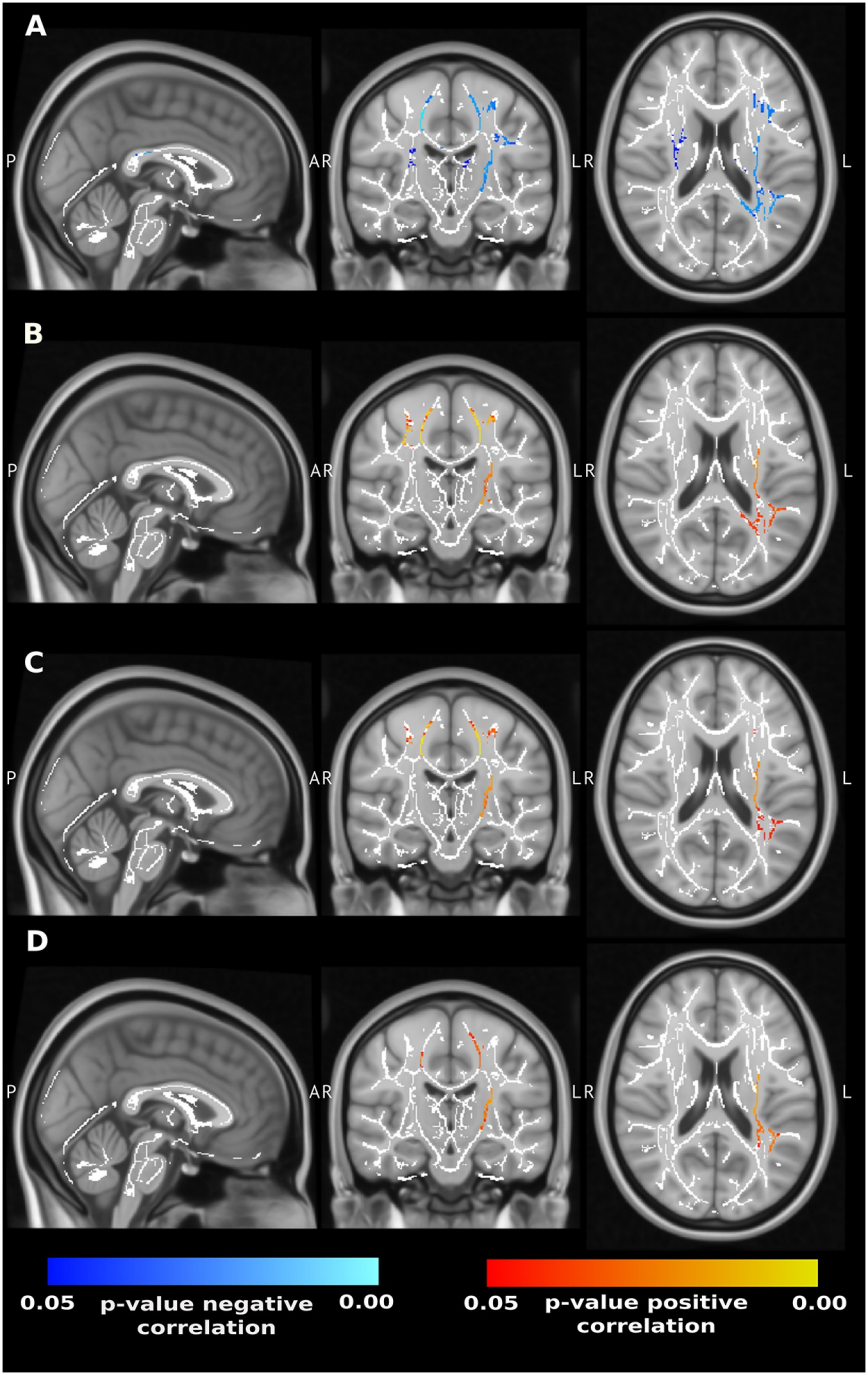
The results of TBSS for fractional anisotropy (FA). Blue colour scale signifies negative correlation between FA and the clinical variable, red colour scale stands for positive correlation (either positive or negative correlation is depicted per clinical scale). **A:** Negative correlation of FA and age. **B:** Positive correlation between FA and executive functions. **C:** Positive correlation between FA and attention. **D:** Positive correlation between FA and memory. Note that the correlations shown in **B**, **C**, **C**, did not persist (as statistically significant) after controlling for age, that was indeed detected as a significant analysis confound, see **A**.

The results of TBSS showed a positive correlation of FA with attention, executive functions, and memory (Fig 3). However, there was also a significant negative correlation with age. Notably, after controlling for this variable, no statistically significant relationship was identified with cognitive scales. In contrast, the position of stroke (in terms of the affected hemisphere) did not play a significant role in the prediction of cognitive status. We consequently disregarded this variable in further analyses. Mostly equivalent results held for the rest of the diffusion metrics. In all cases, we observed widespread negative correlation with executive functions and attention, none of which survived controlling for widespread positive correlation with age (S1–S3 Figs).

In the structural connectivity approach, a similar behaviour occurred. Specifically, we observed one pair of regions significantly correlated with memory, two pairs of regions significantly correlated with attention, and five pairs of regions correlated with visuospatial functions (all significant after the FDR correction). However, the age variable was again predictive of SC. Five pairs of regions showed a significant negative and two pairs significant positive correlation. After controlling for age, we did not observe any link (belonging to the backbone) between the number of tracts and any of the cognitive scales which would survive the FDR correction.

**Fig 4:**
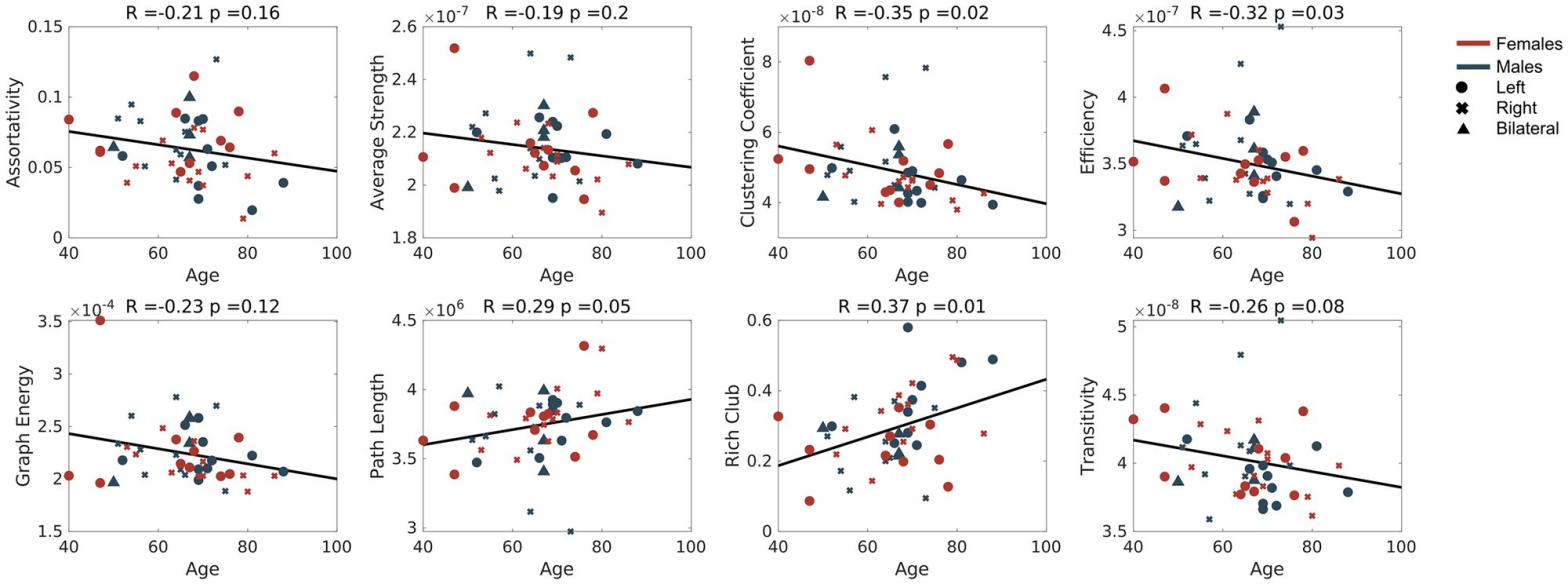
The relationship of age and the graph-related measures. Scatter plots of the graph-theoretical measures computed from the structural connectivity matrices of all subjects. Subjects are visually stratified according to sex (colour) and the lesion location (marker).

In case of graph-theoretical measures, we observed a slightly different behaviour. Relationship between the features and age was less predominant—only clustering coefficient, efficiency and rich club were significantly correlated (p = 0.0313, p = 0.0157, p = 0.0115 respectively, Fig 4). Moreover, none of the clinical scales was correlated with the features even before the age-correction. This suggests that the graph-theoretical measures are less sensitive towards the effect of age that the previous methods, however, also less sensitive to the (future) cognition status.

## Discussion

We examined associations between the condition of white matter acquired shortly after stroke and cognitive status measured with one-year delay. The prolonged period between the two examinations is uncommon in this setup. Thus, rather than reflect an immediate relationship between brain damage and cognition, the potential findings would reflect a predictive character of MRI features on later cognition. In contrast with previous works, we investigated different levels of resolution and methodology, namely: TBSS, statistical analysis of SC matrices, and their graph-theoretical measures. Regardless of the analytical approach, we observed a strong effect of age, which was driving apparent correlations with the clinical scales. In all cases, correlations with clinical scales did not persevere after controlling for age.

### TBSS analysis

The most significant correlate of all WM-derived metrics in the TBSS analysis proved to be age, which was related to a widespread change of diffusion metrics. Additionally, executive functions, attention and memory score were significantly correlated with FA, MD, and RD. Only attention and memory were correlated with AD. However, after controlling for age, the effect in neither of the cognitive scales (and diffusion metric) was preserved. Indeed, cognitive decline is associated with ageing in healthy individuals [65], which suggests the effect of age on cognition would be relevant also in subjects after stroke. However, the effect of age is not always discussed or controlled for in quantitative analyses, risking that its effect might be inappropriately assigned to other variables of interest [66, 67].

We also considered the effect of lesion laterality which was previously identified as an independent predictor of cognitive outcome after stroke [68–71]. However, the affected hemisphere did not play a significant role in the TBSS model and we consequently disregarded it from further analyses. It is probable that the information about position is already intrinsically present in the data in form of reduced FA, alternatively, the (almost) binary nature of the position encoding might not have been precise enough to play a role in the analysis.

Dacosta *et al.* [21] studied 14 patients with right hemispheric stroke and found a significant decrease of FA in right brain anatomical areas compared to healthy controls. They demonstrated a relationship between cognitive functions and FA in several regions in both hemispheres. In another study [23], FA in the thalamus was associated with lower verbal fluency performance. However, in both cases the sample size was relatively small–17 patients in the latter study and subgroups as small as 8 or 6 subjects in the former study and, notably, neither study discussed the effect of age nor reported controlling for it. We did not observe any comparable results in the language or executive functions domain, neither in the whole white matter skeleton analysis, nor in a targeted analysis limiting the region only to the thalamic area as in the original study [23]. Of note, there is a range of relatively smaller differences between the studies, such as that time of acquisition, which was three months after stroke in the prior studies mentioned.

Zamboni *et al.* [72] studied the effect of acute stroke on early cognitive impairment (measured one month after the attack) represented by the Montreal Cognitive Assessment Scale and Mini-Mental State Exam on over 400 patients. In this case, the Montreal Cognitive Assessment Scale was correlated with reduced FA in the anterior tracts after controlling for the Mini-Mental State Exam.

In a longitudinal study following 117 stroke patients, 25 of which were cognitively impaired, lower remote white matter integrity was associated with a worse long-term cognitive performance [20]. This result is specific as it reflects the relationship between cognition and white matter eleven years after the attack.

The reasons behind the absence of effect in the TBSS part of our study may be multifactorial. As argued above, the inconsistent practice concerning controlling for the effect of age may play a role in the heterogeneity of the previously reported results, as well as the time of DTI and cognitive scales assessment. In our case, the scans were taken in the subacute phase–within the second week after the stroke, whereas the cognitive scales were measured one year after, effectively attempting a more challenging medium-term cognitive outcome prediction rather than (almost) instantaneous correlation.

### Structural connectivity analysis

On a structural connectome level we again detected pairs of regions significantly correlated with some of the clinical scales (memory, attention, and visuospatial functions), none of which persisted after controlling for age. We identified five pairs of regions significantly negatively correlated with age after the FDR correction, in line with the intuitive interpretation of the reduction of white matter tracts over time.

Additionally, two pairs of regions were identified to positively correlate with age. This observation is to a degree counterintuitive. However, such increases in strength of shorter tracts might be a technical consequence of an overall white matter deterioration with age. In particular, such deterioration might complicate correct tracing of longer tracts—thus disproportionately increasing the number of short tracts (when working with a fixed amount of tracts). Indeed the two pairs of tracts positively correlated with age in our dataset were on average shorter than those negatively correlated with age.

In related works which investigated SC in relationship to clinical outcome such as aphasia, Yourganov *et al.* [32] constructed connectomes of 90 stroke patients scanned at least six, but on average, 42 months after the attack. The results highlighted the area of temporoparietal junction and its connectivity as essential for language tasks, which was later supported by further analyses [73]. Apparently contradictory are the results from an extensive study that used the methodology of Yourganov *et al.* and acquired data of 818 patients suffering from aphasia approximately 58 months after a stroke [34]. The purpose of the study was to assess the added value of the structural disconnection information on top of the lesion load features to predict language score. No additional effect of the structural information was observed. Nevertheless, the study did not directly use the DTI data to evaluate the structural connectivity but rather imposed the disconnection defined by the lesion location on healthy subjects tractography. This approach might thus disregard the remote structural changes caused by a stroke that would affect language performance.

None of the cognitive scales was significantly related to the structural connectivity in this framework. The reason behind the absence of a relationship may be tied to a common natural issue with such observational studies, that is, missing information on the exact cognitive scores before stroke. Without the reference of the patient’s cognitive performance before the stroke, the specific individual impact of stroke with respect to premorbid cognition can not be exactly inferred—this is a common problem for studies of stroke effects, or other unexpected clinical events. Moreover, the potential presence of small vessel disease or other related conditions [74] could affect both the white matter and cognnitive variables, leading to both spurious positive and false negative results depending on the specific effects.

Finally, in order to more directly connect and compare the results of the TBSS with our findings in structural connectivity, we extracted mean diffusion metrics along the backbone tracts in all patients and performed the same analysis (Spearman correlation with the FDR correction). Our findings further supported the results of TBSS in terms of finding multiple significant correlations with age across backbone tracts whereas finding no correlation with other cognitive scales after controlling for age (S4 Fig). Overall, the effect of age on diffusion and the metrics derived thereof is a widely discussed topic, however, it is still not fully understood. There is sufficient evidence that the diffusion metrics are sensitive to age [75], however, this effect is not necessarily homogeneous across the brain, but spatially varies [76–78]. Consequently, the sensitivity of the DTI-derived measures to age varies as well. In our study AD, MD, and RD appeared to be more sensitive towards the effect of age than FA (in terms of number of connections significantly correlated with age). This is consistent with the results of TBSS, where the effect of age was more widespread for other measures than for the FA.

### Graph-theoretical measures analysis

In the final part of our analysis, the features represented by the graph-theoretical measures were less sensitive towards the effect of age than in the previous approaches. Despite the high degree of intercorrelation between the features, only clustering coefficient, efficiency and rich club coefficient were significantly correlated with age. Additionally, no relationship between the features and clinical scales was found. There have been studies using other graph connectivity measures to study the effects of stroke [79–81]. Among the reported findings were the correlation of The National Institute of Health Stroke Scale with betweenness centrality of the right pallidum and the clustering coefficient of the left superior occipital gyrus, and a positive correlation between the nodal betweenness centrality of the posterior cingulate gyrus and immediate recall [80, 81]. Upon replicating the measures, we did not observe any of the effects above, which, again, may be a consequence of the discrepancies in the designs and cognitive scales used. Notably, compared to the earlier discussed TBSS findings that we have not been able to confirm, these two studies included explicit control for age (as a key potential confound), and had a higher sample size (*N* = 46 and *N* = 15). Apart from some relatively minor technical differences and the ever-present chance of a false positive/negative result, the potential key factor behind the lack of replication of the observation of cognitive correlates of local graph theoretical measures reported by [80] is the temporal difference between the MRI and cognitive assessment–our study attempted one-year prediction, while the previous study apparently works with almost concurrent measurements.

### Limitations

As was pointed out in the earlier parts of the article, the main impediment of our analysis is the lack of premorbid cognition scores for our participants, which affects our ability to unequivocally assign any observed relationship of white matter and cognition score to stroke only. Unfortunately this is the limitation of all studies discussing this topic and may only be solved by designing prospective trial focused on individuals at risk of stroke. More tangible limitation of this work is in the heterogeneity of the lesion location in participants. The inclusion criteria did not specify the position of stroke. Consequently, it is possible that were the lesions locations more consistent across the dataset, more specific conclusions might have been drown. However, to maximize the size of the data, we did not conduct more position-specific analysis. Finally, as the diffusion acquisition protocol was optimized for widespread use in the hospital setup, we were limited by the methods which might be applicable to our data. In our case, we consciously used more conservative methods of fibre tracking and limited the analysis to mostly adjacent pairs of regions and employed rigorous methods of statistical testing to minimize the possibility of obtaining false positive results.

## Conclusion

In this work, we focused on linking the white matter integrity in patients with stroke with the prediction of cognitive status one year after the insult. Using a standard TBSS analysis, we showed that cognitive correlates of white matter fractional anisotropy in patients with stroke can be attributed to the general effect of interindividual age differences—an effect that has not been considered in some previous studies. Indeed, we were not able to reproduce some of the earlier TBSS findings from such analysis of smaller datasets; although this could be also ascribed to the more challenging *forecasting*, rather than *nowcasting* nature of our statistical prediction task. Our subsequent analyses of structural connectivity matrices and graph-theoretical measures further supported our observation of the effect of age driving the correlations with clinical scales. However, the direct interpretability of the structural network predictors of cognitive outcome of stroke is not straightforward and asks for further investigation.

## Supporting information

S1 Table Results of clinical tests across 46 patients. (PDF)

S1 Fig The results of TBSS for axial diffusivity (AD). Blue colour scale signifies negative correlation between AD and the clinical variable, red colour colour scale stands for positive correlation. Either positive or negative correlation is depicted per clinical scale). We observed: **Age:** Widespread positive correlation of AD and age. **Attention:** Global negative correlation between AD and attention. Negative correlation between AD and executive functions. **Memory:** Scattered negative correlation between AD and memory. Localized negative correlation between AD and language. Note that the correlations with clinical scales did not persist (as statistically significant) after controlling for age.
(TIF)

S2 Fig The results of TBSS for mean diffusivity (MD). Blue colour scale signifies negative correlation between MD and the clinical variable, red colour colour scale stands for positive correlation. Either positive or negative correlation is depicted per clinical scale). We observed: **Age:** Widespread positive correlation of MD and age. **Attention:** Global negative correlation between MD and attention. **Executive functions:** Negative correlation between MD and Executive functions. Scattered negative correlation between MD and language. **Memory:** Localised negative correlation between MD and memory. Scattered negative correlation between MD and language. Note that the correlations with clinical scales did not persist (as statistically significant) after controlling for age.
(TIF)

S3 Fig The results of TBSS for radial diffusivity (RD). Blue colour scale signifies negative correlation between RD and the clinical variable, red colour colour scale stands for positive correlation. Either positive or negative correlation is depicted per clinical scale). We observed: **Age:** Widespread positive correlation of RD and age. **Attention:** Negative correlation between RD and attention predominantly in the left hemisphere. **Executive functions:** Scattered negative correlation between RD and executive functions. Scattered negative correlation between RD and Language. **Visuospatial functions:** Localized negative correlation between RD and visuospatial functions. **Memory:** Localised negative correlation between RD and memory. Note that the correlations with clinical scales did not persist (as statistically significant) after controlling for age.
(TIF)

S4 Fig Correlation of fractional anisotropy mean diffusivity, axial, and radial diffusivity with age along the backbone tracts. We extracted tracts between each pair of regions along the backbone and computed average FA and MD. The figures depict the FDR-corrected p-values of Spearman correlations of these values with age. The colour indicates, whether the correlation is positive or negative. Note that no pair of regions was significantly correlated with any diffusivity metrics after controlling for age.
(TIF)
