# Supplementary material for: Structural connectivity-based predictors of cognitive impairment in stroke patients attributable to aging
Source: PLoS One. 2023 Apr 14;18(4):e0280892. doi: 10.1371/journal.pone.0280892 (PMC10104329; doi:10.1371/journal.pone.0280892)
Supplement: S1 Table — (PDF) [file pone.0280892.s001.pdf]

S1 Table: Results of clinical tests across 46 patients.

	**Men mean (sd, min, max)**	**Women mean (sd, min, max)**	**All mean (sd, min, max)**
**Mini-mental state examination**	27.21 (2.5, 18, 30)	27.68 (1.6, 24, 30)	27.43 (2.1, 18, 30)
**FCSRT free recall**	28 (8, 11, 39)	32 (4, 25, 40)	30 (6, 11, 40)
**FCSRT total recall**	47 (1, 43, 48)	48 (1,46,48)	48 (1,43,48)
**FCSRT delayed free recall**	9 (3, 3, 15)	11 (2, 8, 15)	10 (3, 3, 15)
**FCSRT delayed total recall**	16 (0, 15, 16)	16 (0, 15, 16)	16 (0, 15, 16)
**TMT B (time to completion)**	182 (142, 50, 500)	168 (128, 54, 500)	176 (134, 50, 500)
**P-VF**	32 (13, 11, 64)	39 (14, 19, 72)	35 (14, 11, 72)
**Similarities**	22 (5, 8, 29)	20 (5, 10, 28)	21 (5, 8, 29)
**PST - colour (time to completion)**	38 (17, 22, 88)	41 (18, 19, 93)	40 (17, 19, 93)
**TMT A (time to completion)**	51 (26, 23, 123)	43 (15, 20, 68)	47 (22, 20, 123)
**DS forward**	8 (2, 4, 12)	8 (2, 6, 13)	8 (2, 4, 13)
**DS backward**	5 (2, 2, 8)	6 (2, 3, 11)	5 (2, 2, 11)
**BNT-15 (number of errors)**	2 (1, 0, 5)	2 (2, 0, 6)	2 (2, 0, 6)
**C-VF animals**	20 (5, 11, 29)	22 (6, 11, 32)	21 (6, 11, 32)
**ROCF copy**	27 (3, 21, 32)	27 (5, 14, 35)	27 (4, 14, 35)
**VOSP number location**	9 (1, 7, 10)	9 (2, 1, 10)	9 (2, 1, 10)
