# Supplementary figures and images for: Structural connectivity-based predictors of cognitive impairment in stroke patients attributable to aging
Source: PLoS One. 2023 Apr 14;18(4):e0280892. doi: 10.1371/journal.pone.0280892 (PMC10104329; doi:10.1371/journal.pone.0280892)

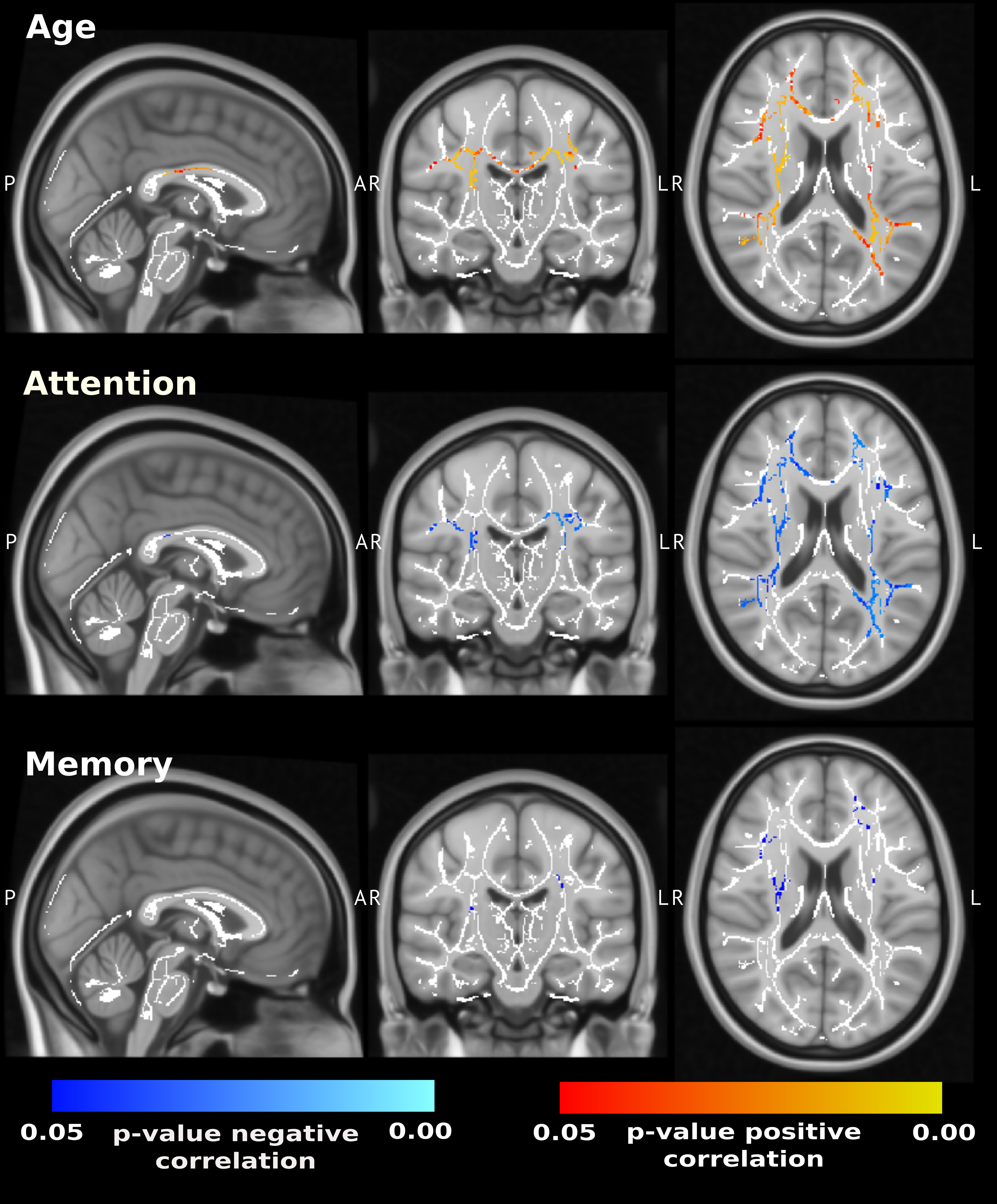

Supplement: S1 Fig — Blue colour scale signifies negative correlation between AD and the clinical variable, red colour colour scale stands for positive correlation. Either positive or negative correlation is depicted per clinical scale). We observed: Age: Widespread positive correlation of AD and age. Attention: Global negative correlation between AD and attention. Negative correlation between AD and executive functions. Memory: Scattered negative correlation between AD and memory. Localized negative correlation between AD and language. Note that the correlations with clinical scales did not persist (as statistically significant) after controlling for age. (TIF) [file pone.0280892.s002.tif]

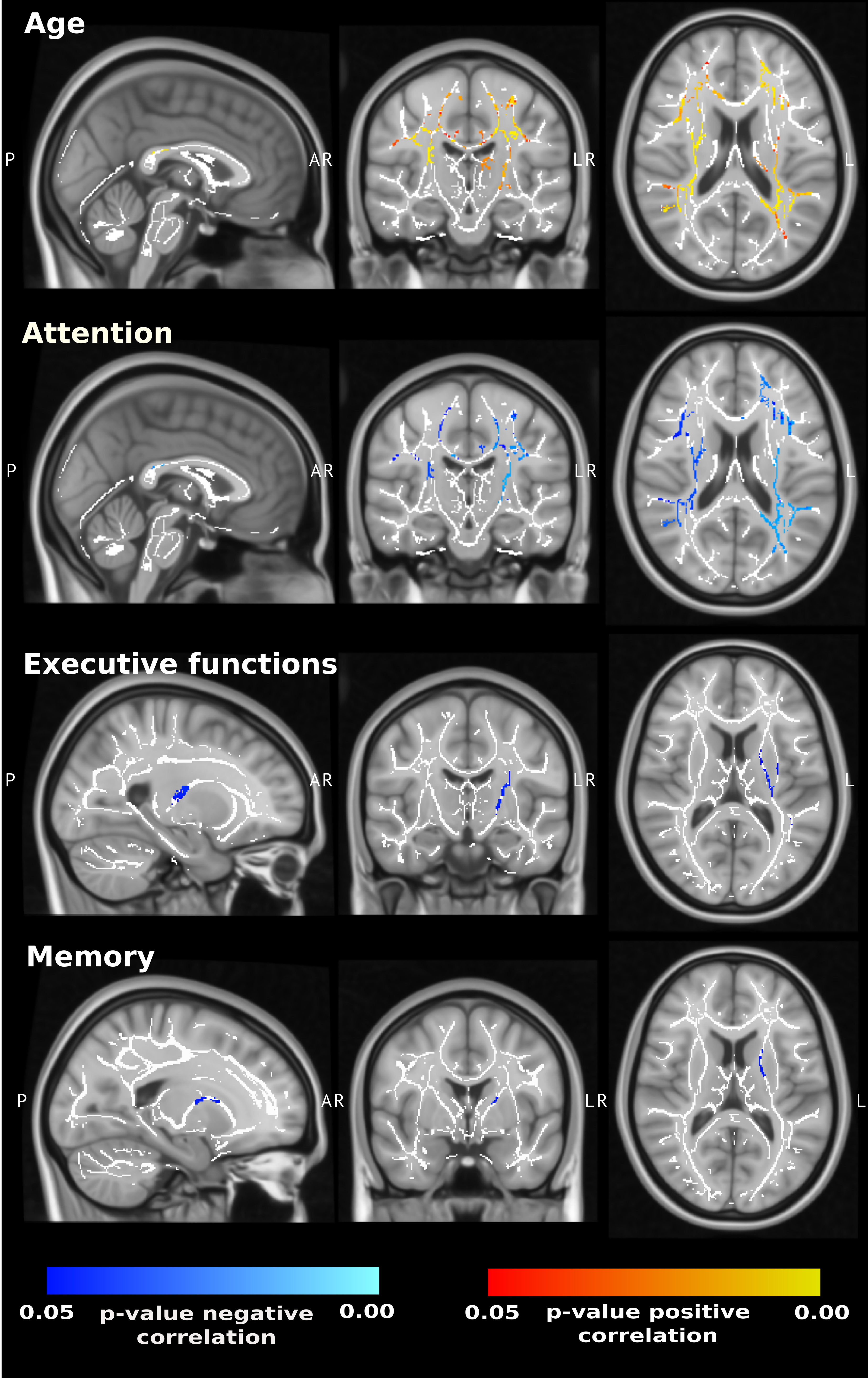

Supplement: S2 Fig — Blue colour scale signifies negative correlation between MD and the clinical variable, red colour colour scale stands for positive correlation. Either positive or negative correlation is depicted per clinical scale). We observed: Age: Widespread positive correlation of MD and age. Attention: Global negative correlation between MD and attention. Executive functions: Negative correlation between MD and Executive functions. Scattered negative correlation between MD and language. Memory: Localised negative correlation between MD and memory. Scattered negative correlation between MD and language. Note that the correlations with clinical scales did not persist (as statistically significant) after controlling for age. (TIF) [file pone.0280892.s003.tif]

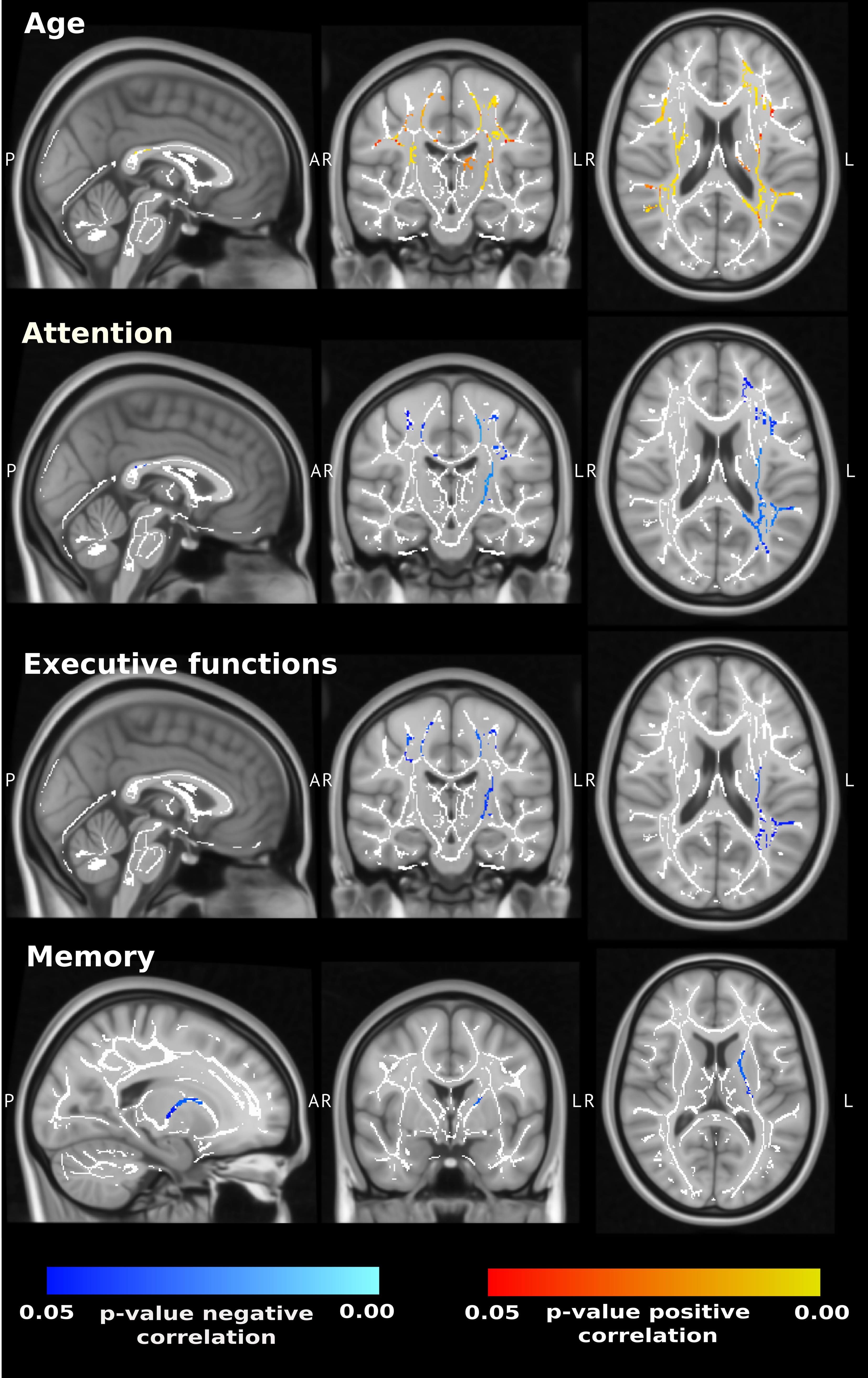

Supplement: S3 Fig — Blue colour scale signifies negative correlation between RD and the clinical variable, red colour colour scale stands for positive correlation. Either positive or negative correlation is depicted per clinical scale). We observed: Age: Widespread positive correlation of RD and age. Attention: Negative correlation between RD and attention predominantly in the left hemisphere. Executive functions: Scattered negative correlation between RD and executive functions. Scattered negative correlation between RD and Language. Visuospatial functions: Localized negative correlation between RD and visuospatial functions. Memory: Localised negative correlation between RD and memory. Note that the correlations with clinical scales did not persist (as statistically significant) after controlling for age. (TIF) [file pone.0280892.s004.tif]

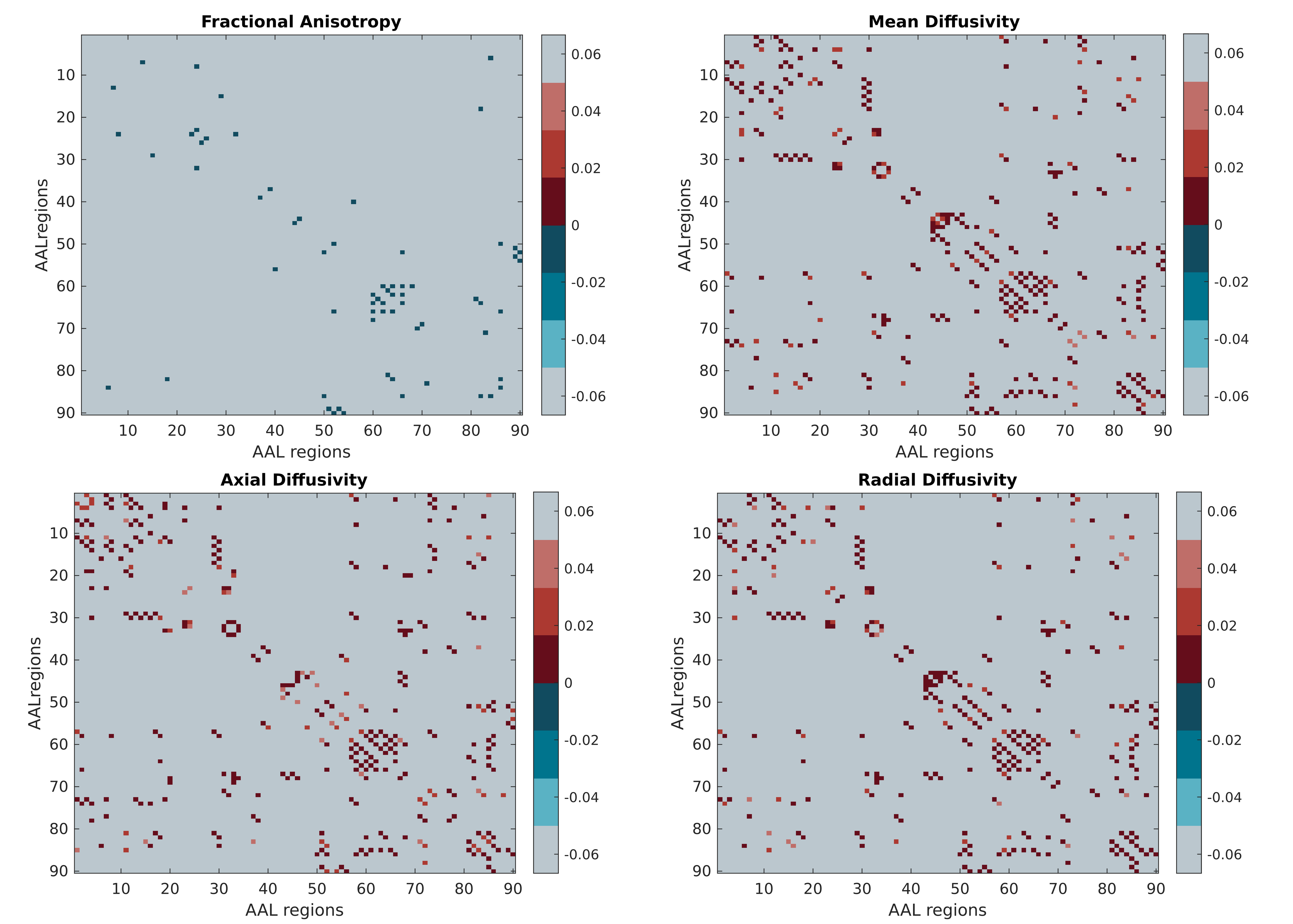

Supplement: S4 Fig — We extracted tracts between each pair of regions along the backbone and computed average FA and MD. The figures depict the FDR-corrected p-values of Spearman correlations of these values with age. The colour indicates, whether the correlation is positive or negative. Note that no pair of regions was significantly correlated with any diffusivity metrics after controlling for age. (TIF) [file pone.0280892.s005.tif]
